# Tissue-Specific Functions of *fem-2*/PP2c Phosphatase and *fhod-1*/formin During *Caenorhabditis elegans* Embryonic Morphogenesis

**DOI:** 10.1534/g3.118.200274

**Published:** 2018-05-02

**Authors:** Osama Refai, Ryan B. Smit, SarahBeth Votra, David Pruyne, Paul E. Mains

**Affiliations:** * Department of Biochemistry and Molecular Biology, University of Calgary, Calgary, Alberta, T2N 4N1, Canada; † Department of Cell and Developmental Biology, State University of NY Upstate Medical University, Syracuse, NY 13210

**Keywords:** *C. elegans*, morphogenesis, epidermis, embryo, formin

## Abstract

The cytoskeleton is the basic machinery that drives many morphogenetic events. Elongation of the *C. elegans* embryo from a spheroid into a long, thin larva initially results from actomyosin contractility, mainly in the lateral epidermal seam cells, while the corresponding dorsal and ventral epidermal cells play a more passive role. This is followed by a later elongation phase involving muscle contraction. Early elongation is mediated by parallel genetic pathways involving LET-502/Rho kinase and MEL-11/MYPT myosin phosphatase in one pathway and FEM-2/PP2c phosphatase and PAK-1/p21 activated kinase in another. While the LET-502/MEL-11 pathway appears to act primarily in the lateral epidermis, here we show that FEM-2 can mediate early elongation when expressed in the dorsal and ventral epidermis. We also investigated the early elongation function of FHOD-1, a member of the formin family of actin nucleators and bundlers. Previous work showed that FHOD-1 acts in the LET-502/MEL-11 branch of the early elongation pathway as well as in muscle for sarcomere organization. Consistent with this, we found that lateral epidermal cell-specific expression of FHOD-1 is sufficient for elongation, and FHOD-1 effects on elongation appear to be independent of its role in muscle. Also, we found that *fhod-1* encodes long and short isoforms that differ in the presence of a predicted coiled-coil domain. Based on tissue-specific expression constructions and an isoform-specific CRISPR allele, the two FHOD-1 isoforms show partially specialized epidermal or muscle function. Although *fhod-1* shows only impenetrant elongation phenotypes, we were unable to detect redundancy with other *C. elegans* formin genes.

Epithelial morphogenesis is a key aspect of animal development, shaping tissues, organs and the organism as a whole (Guillot and Lecuit 2013; Heisenberg and Bellaiche 2013). *C. elegans* embryonic elongation is a simple system to study epithelial morphogenesis. Midway through *C. elegans* embryonic development, the worm embryo undergoes dramatic changes to elongate from a spheroid into a long, thin worm (Sulston *et al.* 1983; Priess and Hirsh 1986; Chisholm and Hardin 2005; Vuong-Brender *et al.* 2016). These morphogenetic events take place without major changes in cell number or extensive cell migration. The embryo reduces its circumference by a factor of three and increases in length by a factor of four during the elongation process. Embryonic elongation can be divided into two main phases, early and late elongation. Early elongation occurs between the 1.2 and 2-fold stages (“fold” refers to the length of the embryo relative to the long axis of the eggshell) while late elongation occurs after 2-fold. Early elongation is mediated by cell shape changes in the single cell layer that surrounds the embryo, the epidermis (also referred to as the hypodermis in nematodes). Epidermal actomyosin contractility provides the main force that mediates early elongation (Priess and Hirsh 1986; Chisholm and Hardin 2005; Vuong-Brender *et al.* 2016). Late elongation depends on muscle cell contraction (Williams and Waterston 1994; Zhang *et al.* 2011).

Two parallel pathways mediate early elongation (Wissmann *et al.* 1997; Piekny *et al.* 2000; Gally *et al.* 2009; Martin *et al.* 2014). One pathway involves Rho-binding kinase (ROCK, LET-502 in *C. elegans*), which activates non-muscle myosin II (NMY-1/NMY-2) by phosphorylating the regulatory myosin light chain (MLC-4, Figure 1). This activation is antagonized by a corresponding PP1c myosin phosphatase. However, activated ROCK/LET-502 also inactivates myosin phosphatase’s brake to contraction by phosphorylating the myosin phosphatase MYPT targeting subunit (MEL-11). Loss of *let-502* thus results in hypocontraction and elongation arrest at the 1.2-fold stage. Conversely, mutations of *mel-11*, the inhibitor of contraction, result in hypercontraction and embryos often burst (Wissmann *et al.* 1997; Wissmann *et al.* 1999; Diogon *et al.* 2007). However, *let-502*; *mel-11* double mutant embryos often elongate and grow to adulthood, albeit animals are sterile with abnormal body shapes (Wissmann *et al.* 1997). This indicates the presence of a parallel pathway, which includes p21 activated kinase/PAK-1 and its regulators PP2c phosphatase/FEM-2 and the CDC42/RAC-specific guanine-nucleotide exchange factor (GEF)/PIX-1 (Piekny *et al.* 2000; Diogon *et al.* 2007; Vanneste *et al.* 2013; Martin *et al.* 2014). PIX-1 and PAK-1 appear to be more active in the anterior of the embryo and the Rho and Rac pathways institute differing morphogenetic programs (Martin *et al.* 2016; Martin *et al.* 2017). Redundancy between the two parallel pathways is detected by strong enhancement of elongation defects seen when mutations of the different pathways are combined in double mutants.

**Figure 1:**
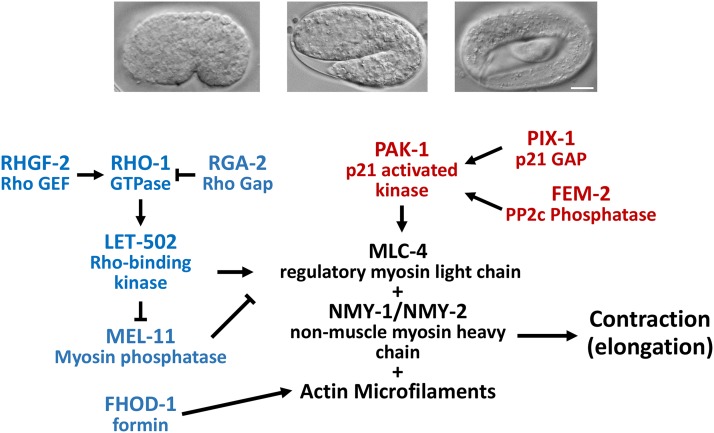
Model of early *C. elegans* embryonic elongation. Top, photomicrographs of 1.2 fold, 1.7-fold and 4-fold embryos, scale bar = 10 µm. Bottom, actomyosin contractility is triggered by phosphorylation of MLC-4/regulatory myosin light chain and inhibited by MEL-11/myosin phosphatase. Phosphorylation is mediated by kinases in two parallel pathways, PAK-1/p21 activated kinase (red; dorsal/ventral epidermal cells) and LET-502/Rho-binding kinase (blue; lateral epidermal cells). The actin nucleator FHOD-1/formin genetically acts in the LET-502 pathway.

Prior to the start of embryonic elongation, epidermal cells migrate to encase the entire embryo (Sulston *et al.* 1983; Williams-Masson *et al.* 1997). These cells are organized in three rows: (1) one row of epidermal cells straddling the dorsal midline along the anterior-posterior axis; (2) two rows (one on each side of the embryo) of lateral epidermal cells (also known as seam cells); and (3) two rows of ventral epidermal cells on each side of the embryo meeting at the ventral midline. The epidermal cell rows show different behaviors during early elongation as the lateral cells show stronger actinomyosin contractility while the dorsal/ventral cells play a more passive role (Priess and Hirsh 1986; Diogon *et al.* 2007; Gally *et al.* 2009; Vuong-Brender *et al.* 2017). This is reflected in the epidermal cytoskeleton. Actin microfilaments and microtubules become organized in circumferential patterns within the dorsal and ventral epidermal cells, but actin filaments and microtubules remain as a meshwork in the contractile lateral epidermal cells until the 2-fold stage (Priess and Hirsh 1986; Gally *et al.* 2009). After early elongation is completed, microfilaments in the lateral cells align along the circumferential axis. A number of components are implicated in this asymmetric behavior between the lateral or dorsal/ventral epidermis with contractility functions being enriched laterally (Figure 1). *let-502* transcriptional reporters show higher expression in the lateral compared to dorsal/ventral cells with *mel-11* showing the opposite pattern (Wissmann *et al.* 1999). The Rho activating guanine exchange factor (GEF/RHGF-2) and Rho inhibiting GTPase activating protein (GAP/RGA-2) are both expressed throughout the epidermis. However, lateral expression of RHGF-2 and dorsal/ventral expression of RGA-2 are each sufficient for embryonic elongation (Diogon *et al.* 2007; Chan *et al.* 2015). PIX-1 and PAK-1 act in the dorsal epidermis (Martin *et al.* 2014; Martin *et al.* 2016).

FHOD-1, which belongs to the formin family of proteins with nucleating and bundling functions, may also contribute to lateral *vs.* dorsal/ventral differences. During *C. elegans* elongation, *fhod-1* mutants disrupt microfilament organization in the contractile lateral epidermis and result in early elongation arrest. However, these are low penetrance phenotypes, perhaps suggesting redundancy with other formins or actin organizing factors. *fhod-1* genetically acts downstream of *let-502/mel-11* and in parallel to *pak-1/fem-2* (Figure 1) (Vanneste *et al.* 2013). Consistent with *fhod-1* acting with *let-502/mel-11* but not *pix-1/pak-1*, FHOD-1 shows strongest expression in the lateral epidermis, where *let-502/mel-11* appears to act. However, FHOD-1 antibody staining is detected late in elongation, even though genetic effects are seen during early elongation.

Members of the formin protein family function during actin-mediated processes by influencing the assembly and elongation of unbranched actin filaments (Breitsprecher and Goode 2013; Bechtold *et al.* 2014; Kuhn and Geyer 2014; Pruyne 2016). Family members nucleate microfilaments and then bind to the growing (barbed) ends as a progressive cap that prevents filament termination by capping proteins. Formins have characteristic formin homology (FH) domains. FH1 recruits profilin-bound actin, FH2 is involved in formin dimerization, and the FH3/Diaphanous Inhibitory Domain/DID interacts with the C-terminal Diaphanous Autoregulatory Domain/DAD domain to cause autoinhibition. Formins can be activated by binding a Rac GTPase in a nucleotide-independent manner or by phosphorylation of the DAD domain by Rho-binding kinase (although the latter does not appear to be essential in *C. elegans* (Vanneste *et al.* 2013)). A coiled-coil domain is often found between the FH1 and FH3 domains. In addition to their role in actin modulation, formins have also been implicated in microtubule dynamics (Breitsprecher and Goode 2013; Kuhn and Geyer 2014).

*fhod-1* is the sole *C. elegans* member of the FHOD subfamily of formins and is related to the human and mouse FHOD1 and FHOD3 (Mi-Mi *et al.* 2012; Pruyne 2016). FHODs are involved in stress fiber formation, cell spreading and adhesion, and myofibril maturation (Taniguchi *et al.* 2009; Iskratsch *et al.* 2010; Kan-O *et al.* 2012a; Breitsprecher and Goode 2013; Bechtold *et al.* 2014). In addition to actin assembly and stress fiber formation, FHOD bundles microfilaments *in vitro* (Alvarez and Agaisse 2013; Schonichen *et al.* 2013; Patel *et al.* 2018). FHOD-1 also functions in *C. elegans* muscle, where it is partially redundant with CYK-1 for formation of striated body wall sarcomeres (Mi-Mi *et al.* 2012; Mi-Mi and Pruyne 2015). FHOD-1 is localized near the Z lines (where the actin thin filaments are anchored into the plasma membrane) within the contractile lattice, and along the edges of growing body wall muscle cells. Actin thin filaments still form in *fhod-1* mutants, but muscles are narrower and further apart, and Z lines attachments are defective. Finally, *fhod-1* strongly enhances the spermathecal ovulation defects of the inverted formin *exc-6/inft-1*. The interaction may be indirect, with FHOD-1 acting in egg-laying muscles (Hegsted *et al.* 2016).

The *C. elegans* genome encodes six other formins that could potentially act in concert with *fhod-1*. In addition to the muscle, *cyk-1* acts during embryonic cytokinesis and in the excretory cell (Severson *et al.* 2002; Mi-Mi *et al.* 2012; Shaye and Greenwald 2016). *daam-1* and *frl-1* participate in Wnt-mediated cell polarity of the B cell (Wu and Herman 2006). *fozi-1* encodes a divergent formin required in cell fate specification of muscles and neurons (Johnston *et al.* 2006; Amin *et al.* 2007). In addition to the spermatheca, *exc-6* functions in the excretory cell, as does the second inverted formin, *inft-2* (Shaye and Greenwald 2015; Hegsted *et al.* 2016; Shaye and Greenwald 2016).

In this work, we address several aspects of elongation, including how *fem-2* and *fhod-1* contribute to the contractile differences between lateral and dorsal/ventral cells. We report that *fem-2* expression in the dorsal/ventral epidermis is sufficient for elongation while *fhod-1* acts laterally. We found that *fhod-1* encodes two isoforms that differ in a predicted coiled-coil domain. These appear to have functions specialized for embryonic elongation or adult muscle differentiation, but there is a partial overlap between their functions. The impenetrant phenotype of *fhod-1* mutants could indicate redundancy with other factors, but we were unable to detect genetic interactions with other *C. elegans* formins or actin nucleators. Finally, we examined whether muscles function in early elongation, in addition to their essential role later in the process. PAK-1 and PIX-1 have functions in the hemidesmosomes that link muscle to epidermis (Zhang *et al.* 2011), and FHOD-1 also has roles in embryonic muscle development (Mi-Mi *et al.* 2012; Mi-Mi and Pruyne 2015). Thus, it was theoretically possible that muscles may play a redundant role during early elongation, in parallel with the LET-502 pathway. We found this not to be the case.

## Materials And Methods

### Strains and alleles

*C. elegans* (var. Bristol) were maintained on NGM plates at 15° as described by Brenner (1974) unless otherwise specified. Temperature-sensitive (*ts*) strains were upshifted to 20°, 24° or 25° at the last larval (L4) stage. Complete broods from ≥ 4 hermaphrodites with a total of >100 offspring are reported. The following alleles were used. Descriptions can be found at Wormbase. Linkage Group I: *let-502(sb118ts)*, *fhod-1(tm2363* and *sb123)*, *wve-1(ok3308)*. Linkage Group II: *mel-11(it26ts)*. Linkage Group III: *fem-2(b245ts)*, *frl-1(ok460)*, *exc-6(gk386)*, *fozi-1(ok1182)*, *cyk-1(or36)*, *pat-4(st551)* (rescued by *zpEx184[myo-3*::*pat-4(+)]*). Linkage Group IV: *wsp-1(gm324)*. Linkage Group V *inft-2(ok1296)*, *daam-1(tm2133)*. Alleles were often linked to visible morphological markers to facilitate strain construction and the following balancers were also used: *hT2[bli-4(e937) let(q782) qIs48] (I;III)*, *mnC1[dpy-10(e128) unc-52(e444)] II*, *qC1[dpy-19(e1259) glp-1(q339)] III*, *mls10[myo-2:gfp] V*. Transgenic strains are listed in Table S1.

### Scoring of elongation phenotypes

Elongation defects were examined using a dissecting stereomicroscope with twice the egg length being used to judge 2-fold arrest. Elongation arrest phenotypes were categorized into four groups, (i) unhatched embryos, (ii) early larval arrest, where embryos hatch but elongation arrests at or before the 2-fold stage, (iii) later larval arrest, in which embryos pass the 2-fold stage but usually arrest with a dumpy (Dpy) phenotype, and (iv) growth to L4/adulthood. Screening for redundancy between formins was carried out by RNA interference (RNAi) by feeding (Kamath *et al.* 2003). RNAi clones (www.lifesciences.sourcebioscience.com) were confirmed by sequencing. Three L3 or L4 larvae were transferred to the RNAi plate and subsequently transferred to a fresh RNAi plate every 12 hr. After removing the hermaphrodites, the brood on the third plate was scored, as RNAi should have taken effect. The plate was scored 24 and 48 hr after hermaphrodite removal to assess hatching and growth to L4/adult, respectively. All analyses were carried out at 25°, except as noted.

Transgenic rescue was assessed by measuring worm lengths. L4 hermaphrodites were shifted to the appropriate temperature overnight, and the now gravid animals were transferred to new plates to lay eggs for 2-3 hr. Progeny were photographed 45-48 hr later with a dissecting microscope and lengths were measured using Image J.

### Isolation of fhod-1(sb123) deletion in exon 8

The long isoform of *fhod-1* (*C46H11.11*; WormBase version WS262) includes the 474 bp exon 8, but not the short isoform. The WormBase gene model includes additional exons, but for simplicity, our numbering will only include those that we confirmed by sequencing. To create a *fhod-1* allele defective for the long isoform, we used CRISPR to create an out of frame deletion in this exon. *eft-3*::*Cas9*, pJA58[*dpy-10(cn64)* gRNA] and a *dpy-10(cn64)* repair single-stranded oligonucleotide (University of Calgary Core DNA Services) were used as described for “Co-CRISPR” (Arribere *et al.* 2014). The *fhod-1* exon 8 gRNA PCR product was created using PCR “stitching”. PCR’s on pJW1285 (*pha-1* gRNA(F+E), a gift from Jordan Ward, Addgene) using oJW1787 + exon8rev and oJW1790 + exon8fwd were stitched together, substituting *fhod-1* exon 8 sequences for those of *pha-1* and gel-purified (oligonucleotides are listed in Table S2). Gravid wild-type hermaphrodites were injected with 50 ng/μL *eft-3*::*Cas9*, 25 ng/μL pJA58(*dpy-10(cn64)* gRNA), 500 nM *dpy-10(cn64)* oligonucleotide (Arribere *et al.* 2014) and 25 ng/μL *fhod-1* exon 8 gRNA. Dpy and Rol F1 progeny were screened by PCR and polyacrylamide gel electrophoresis for deletions. A 7 bp deletion was confirmed by sequencing and out-crossed to remove the *dpy-10* co-CRISPR allele.

### Immunostaining and microscopy

For DIC microscopy, hermaphrodites were moved to fresh plate and allowed to lay eggs for a few hours. Embryos were mounted with a drop of M9 buffer on a 2% agar pad (Sulston *et al.* 1983). Embryos were examined with a Carl Zeiss Axio Imager 2 microscope and images were taken by AxioCam camera collected with Axiovision software (Zeiss).

For measurement of body-wall muscle (BWM) and body dimensions, adult worms were stained for F-actin with Alexa 568-phalloidin as described previously (Mi-Mi *et al.* 2012). For each strain, the lateral width of phalloidin-stained BWM, the gap width between BWM quadrants, the number of oblique striations per BWM cell, and the width of the body were analyzed in three replicates of 20 animals each, as described previously (Mi-Mi *et al.* 2012). L4 worms were stained for α-actinin and for FHOD-1 using monoclonal MH35 and polyclonal DPMSP2 antibodies, respectively, as previously described (Mi-Mi *et al.* 2012). All images were acquired at room temperature on an Eclipse 90i upright microscope (Nikon) through a CFI Plan Apochromat 40x/NA 1.0 oil immersion objective with a digital monochrome charge-coupled device camera (Cool-SNAP HQ2; Photometrics) driven by NIS-Elements AR software (version 3.1; Nikon). Measurements of dimensions were performed using NIS-Elements AR. Images processed linearly through Photoshop CS4 (Adobe) to enhance contrast.

### Isolation of RNA and RT-PCR

Gravid hermaphrodites were treated with alkaline hypochlorite using standard protocols and embryos were collected using a sucrose suspension. Total RNA was isolated from both gravid adults and embryos using the TRIzol Plus RNA Purification System (Invitrogen). Reverse transcription PCR (RT-PCR) was carried out using the Pure High Capacity cDNA Reverse Transcription Kit (Applied Biosystems). Two sets of primers were designed at the N-terminus and C-terminus of *fhod-1* cDNA (Nterm-F/R and Cterm-F/R, Table S2). To clone the full cDNA of *fhod-1*, we used PCR primer sets that covered the length of the predicted cDNA (FhdNR.3, Shrt.1F/R, Shrt.2F/R). Embryonic lysate was tested for expression of adult-specific *vit-2* to assess contamination from adult RNA (Vit2-F/R) (Heine and Blumenthal 1986). PCR products of the appropriate size were extracted from gels and were sequenced to confirm their identity. Sequence alignments of FHOD proteins from different organisms were carried out using ClustalW2. Protein domain predictions were carried out using InterPro tool EMBL-EBI and the COILS program (Lupas *et al.* 1991).

### fem-2 and fhod-1 tissue-specific rescue

Tissue-specific expression constructs used information from Wormbase WS244 data freeze. Primers for cloning of the *fhod-1*, *fem-2*, *elt-3*, *ceh-16* and *myo-3* promoters, all with the *unc-54* 3′UTR, are shown in Table S2. The *fem-2* cDNA pDP#DH014 plasmid (Hansen and Pilgrim 1998) was provided by D. Pilgrim. The full-length cDNA was amplified by PCR using the two primers *fem*2-F and *fem*2-R, which introduce the cut sites of *PstI* and *NotI*, respectively. The PCR product was digested with *PstI/NotI* and then ligated into pBS II (SK+) previously digested with same enzymes. The *3′ UTR* of *unc-54* was cloned from wild-type genomic DNA using unc54-F and unc54-F primers, which introduce *NotI* and *SacII* restriction sites, respectively, and introduced to the 3′ end of the *fem-2* cDNA in the pBS II (SK+) vector. Promoters of *fem-2* (femPr-F/R, 344 bp), *elt-3* (elt3-F/R, ∼1.9 kb), *ceh-16* (cehl6-F/R, ∼2.9 kb) and *myo-3* (myo3-F/R, ∼1.9 kb) were amplified by PCR from genomic DNA and digested by *KpnI* and *PstI*, which cut sites introduced by the two primer sets. These were ligated separately into the pBS II (SK+) vector that had the *fem-2* cDNA and *unc-54* 3′ UTR. Each of the four constructs was injected into the *fem-2(b245)*; *fhod-1(tm2363)* (HR1438) strain at 2 ng/μL, together with transformation markers of 20 ng/μL of the gut-specific reporter marker (*elt-2*::*GFP*::*LacZ)* (Fukushige *et al.* 1998), 50 ng/μL of the dominant Roller marker pRF4 (*rol-6(su1006)*) and 48 ng/μL pBS II (SK+). For every *fem-2* cDNA plasmid, two independent transgenic lines were isolated, which are listed in Table S1.

*fhod-1a(long)* and *fhod-1b(short)* cDNAs (with and without exon 8, respectively) were isolated and cloned into pBS KS+ with the *unc-54* 3′UTR as described above. The native *fhod-1* promoter (∼5.3 kb) was isolated from wild-type genomic DNA using oRS63 + oRS70. The *ceh-16* and *elt-3* promoters were isolated using restriction enzyme digestions from *ceh-16*::*rhgf-2*::*unc-54* and *elt-3*::*rhgf-2*::*unc-54* plasmids respectively (Chan *et al.* 2015). The *myo-3* promoter was isolated from wild-type genomic DNA as described earlier for *fem-2*. Rescue plasmids were injected at 2 ng/μL with 50 ng/μL pRF4 (*rol-6(su1006)*) (Mello *et al.* 1991). Lines *sbEx242* through *sbEx249* also included 45 ng/μL pTG96 (*sur-5*::*gfp*) (Gu *et al.* 1998), which marks transgenic worms as GFP+ in all (or most) cells. Constructs were injected into *fhod-1(tm2363) let-502(sb118) I* (HR 1485). Transgenic strains are listed in Table S1.

### Western blot analysis of FHOD-1

For each strain analyzed, mixed-age worm populations were washed from 6 un-starved 60-mm NGM plates using M9 buffer/0.1% triton X-100. The samples were enriched for adult worms by transferring them to 1.7-mL tubes and for four times, allowing the heavier adults to settle by gravity on ice for 2 min before removal of buffer. Adult worms were suspended with 2 volumes 2X SDS-sample buffer, boiled for 3.5 min, disrupted for 30 sec using a tissue grinder, and boiled 3 more minutes. Boiled extracts were drawn 8 times through an insulin syringe to shear genomic DNA before loading for SDS-PAGE. Extracts were normalized and probed for the FHOD-1 FH2 domain using the antibody DPMSP2 as described previously (Mi-Mi *et al.* 2012).

### Data availability

Strains and plasmids are available upon request. The authors affirm that all data necessary for confirming the conclusions of this article are represented fully within the article and its tables and figures. Supplemental material available at Figshare: https://doi.org/10.25387/g3.6199286.

## Results

### fem-2 expression in dorsal and ventral cells can mediate elongation

**Figure 2:**
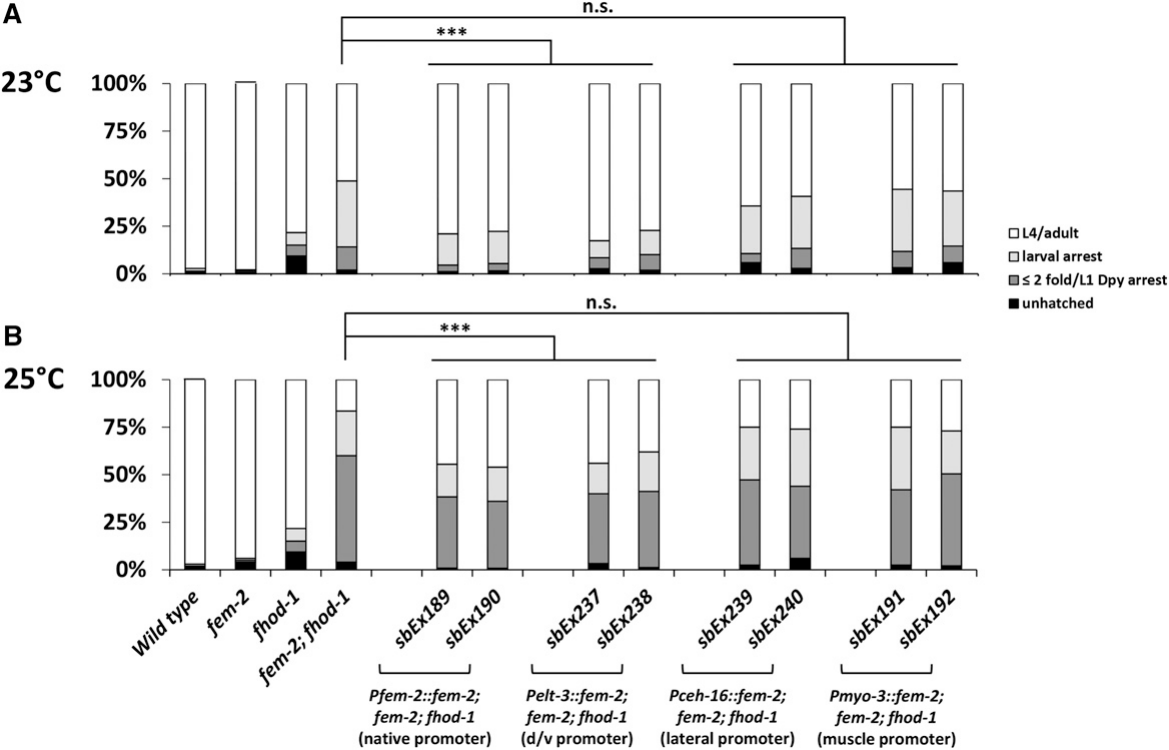
Tissue-specific rescue of *fem-2*. Extrachromosomal arrays contained constructs of the *fem-2* cDNA driven by the *fem-2* promoter, the *elt-3* promoter (dorsal/ventral epidermal cells), the *ceh-16* promoter (lateral epidermal cells) or the *myo-3* promoter (body wall muscle). Two independent lines for each construct were cultured at 23° (A) or 25° (B) and scored for growth to the indicated stages as described in MATERIALS and METHODS. As *fem-2* elongation phenotypes are low penetrance, *fhod-1(tm2363)* was included to sensitize the strains. At both temperatures, the native and dorsal/ventral promoters rescued elongation phenotypes to similar degrees (*** = *P* < 0.001 by One Way ANOVA of the animals that grew to L4/adult) while the lateral and muscle promoters did not (n.s. = non-significant, *P* > 0.05).

In addition to its well-established function in sex determination (Pilgrim *et al.* 1995; Chin-Sang and Spence 1996), *fem-2*/PP2c phosphatase acts during embryonic elongation, in parallel to *fhod-1* and the *mel-11/let-502* pathways (Figure 1) (Piekny *et al.* 2000; Vanneste *et al.* 2013). Other genes that genetically act in the *fem-2* branch of the elongation pathway, including *pak-1* and its activator *pix-1*, act in dorsal/anterior epidermis during elongation (Martin *et al.* 2014; Martin *et al.* 2016). To determine if *fem-2* acts similarly, we expressed a *fem-2* cDNA using drivers that are specifically active in lateral cells (*ceh-16*) or dorsal/ventral cells (*elt-3*). These promoters are expressed before elongation begins (Gilleard *et al.* 1999; Cassata *et al.* 2005; Diogon *et al.* 2007; Gally *et al.* 2009; Chan *et al.* 2015; Quintin *et al.* 2016). Transgenes of these constructs were compared to the same *fem-2* cDNA driven by its native promoter, which is able to rescue the *fem-2* sex determination phenotype (Pilgrim *et al.* 1995; Hansen and Pilgrim 1998). *fem-2* mutant alleles have a low penetrance elongation phenotype (Figure 2) (Piekny *et al.* 2000), with a frequency of < 5%; however, the elongation phenotype for the *ts* allele *fem-2(b245)* can be sensitized in double mutants with *fhod-1* (Vanneste *et al.* 2013). This *fem-2*; *fhod-1* has elongation defects of 50% at 23° and 80% at 25° (Figure 2A,B). Since *C. elegans* transgenes are typically not expressed maternally (Kelly *et al.* 1997) and *fem-2* elongation defects show both maternal and zygotic effects (Piekny *et al.* 2000), we confirmed that zygotic expression of *fem-2(+)* can rescue *fem-2*; *fhod-1* elongation defects (Figure S1A).

*fem-2* expression appears to be sufficient in the dorsal/ventral epidermis for elongation. Animals were transferred daily until they ceased laying embryos, the hatching rate was scored a day after removal of hermaphrodites, and growth was assessed under the dissecting scope the following day. Both *Pfem-2*::*fem-2* and *Pelt-3*::*fem-2* partially rescued elongation defects at 23°. The proportion of worms growing to the L4/adult stage, which includes the rescued animals, increased from 51% in the *fem-2*; *fhod-1* to 79% and 80% for *Pfem-2*::*fem-2* and *Pelt-3*::*fem-2*, respectively (Figure 2A). Similar results were seen at 25°, where the proportion of L4/adults was elevated from 16% in controls to 45% and 43% in *Pfem-2*::*fem-2* and *Pelt-3*::*fem-2* transgenic strains, respectively (*P* ≤ 0.001). In contrast, introducing *Pceh-16* (lateral) and *Pmyo-3* (muscle) constructs driving the *fem-2* cDNA had no significant effect in the elongation phenotype at either temperature (*P* > 0.05). We conclude that dorsal/ventral expression of *fem-2* is at least partially sufficient for successful elongation, consistent with the dorsal/ventral functions observed for *pak-2* and *pix-1*.

### fhod-1 encodes multiple isoforms

**Figure 3:**
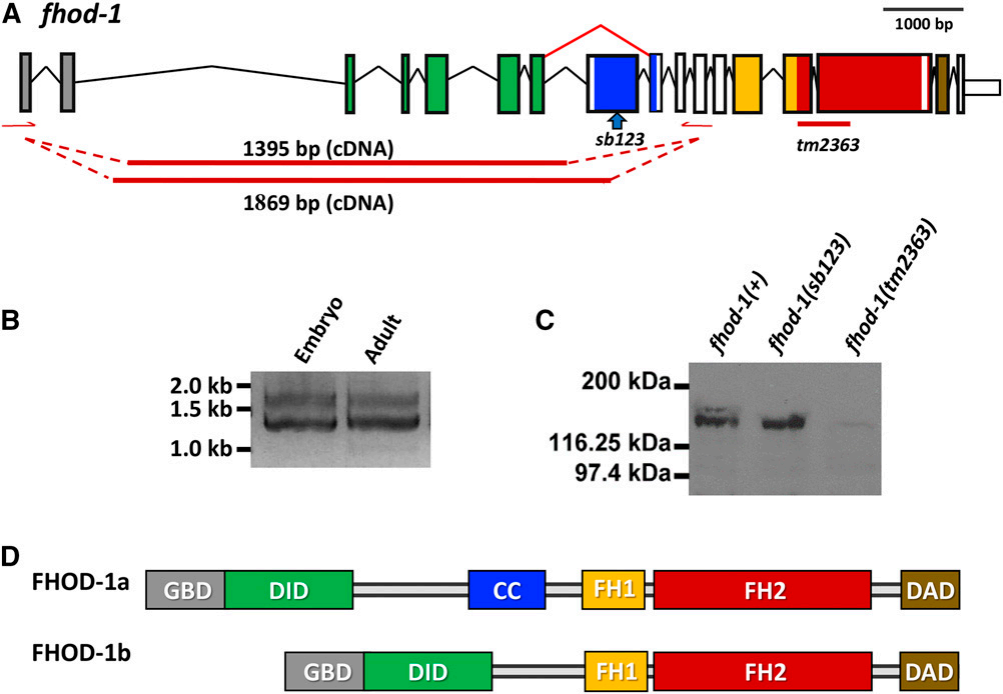
*fhod-1* gene structure and expression. (A) Diagram of the intron/exon structure of the *fhod-1* gene. Exons are color coded to correspond to the protein domains shown in (D). Positions of the *sb123* 7 bp deletion and the 367 bp deletion *tm2363* are indicated. The primers (red arrows) for RT-PCR (B) detected 1.4 kb and 1.8 kb bands in both gravid adults and embryos. Sequencing showed they differed by the inclusion of exon 8 in the longer form. (C) Western blots revealed two species of approximately 173 kD and 158 kD in *fhod-1(+)*. The upper band was missing in *fhod-1(sb123)*, which creates a frameshift mutation in exon 8. Both isoforms are absent from the out of frame deletion allele *tm2363*. (D) Domain structure of the long and short isoforms FHOD-1a and FHOD-1b, respectively, resulting from alternative splicing of exon 8. GBD, Guanine nucleotide binding domain, DID, diaphanous inhibitory domain, CC, predicted coiled-coil, FH1, formin homology domain 1, FH2, formin homology domain 2, DAD, diaphanous autoregulatory domain. The WormBase gene model includes additional exons, but for simplicity, our numbering will only include those that we confirmed by sequencing.

We next examined the role of *fhod-1* during *C. elegans* elongation. We detected two major RT-PCR products for *fhod-1* in both the adult and embryonic total RNA (Figure 3A,B). Sequencing showed that the shorter product lacked 474 bp encoded by exon 8 (WormBase WS262 indicates additional exons, but for simplicity, our numbering only includes those exons that we detected). The two predicted proteins shown in Figure 3 differ by 158 amino acids, corresponding to products of 1346 (FHOD-1a) and 1188 (FHOD-1b) amino acids, and the expected proteins are detected by Western blot (Figure 3C,D). Although conservation of the primary sequence outside nematodes is limited, the region unique to the *C. elegans* long form includes an 83 amino acid predicted coiled-coil region that is similarly predicted in FHOD-1 orthologs in human, *Drosophila*, and *C. briggsae* (Figure S2). Two other *fhod-1* regions have shorter predicted coiled-coils that are common to both *C. elegans* isoforms, including 29 residues between positions 309 and 337 (exon 7) and 22 amino acids from 1085 and 1106 (exon 15). These are also found in the other species.

### Differential functions of long and short fhod-1 isoforms during elongation

To investigate differing functions of *fhod-1a* and *fhod-1b*, we generated *fhod-1(sb123)*, a 7 bp CRISPR deletion allele in the alternatively spliced exon 8. This results in an out of frame product, creating a stop codon after 26 amino acids. If translated, the predicted protein would lack the FH1, FH2 and DAD domains. Western blots demonstrated that the longer isoform is absent from mixed stage *fhod-1(sb123)* while the abundance of the short form was not altered (Figure 3C,D). Thus *sb123* likely represents a null or strong loss of function specific for *fhod-1a* but would retain *fhod-1b(+)* function.

**Figure 4:**
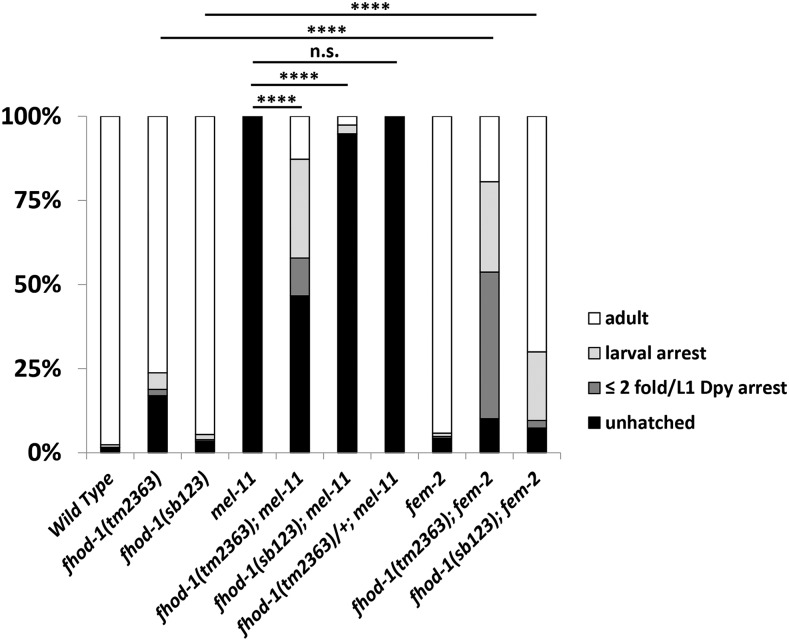
*fhod-1(sb123)*, which encodes only the short isoform, has little effect on elongation in *mel-11* or *fem-2* backgrounds. Indicated genotypes were scored for growth to the indicated stages as described in MATERIALS and METHODS. While the *fhod-1(tm2363)* null allele rescues *mel-11* lethality at 20°C, *sb123* does so weakly and *tm2363/+* does not at all. **** = *P* < 0.0001 and *P* > 0.99 respectively, by One Way ANOVA of hatching rates. Likewise, at 25° *tm2363* enhances *fem-2* elongation defects, while effects of *sb123* are milder. **** = *P* < 0.0001 by One Way ANOVA of growth to L4/adulthood.

We compared animals lacking *fhod-1a* to loss of both *fhod-1* isoforms in the *tm2363* allele. *fhod-1(tm2363)* is an out of frame deletion of the FH2 domain that affects both isoforms and has little or no protein by immunofluorescence (Mi-Mi *et al.* 2012) and lacks both isoforms by western blot (Figure 3A,C). This allele has impenetrant elongation defects with 19% showing arrest by the 2-fold stage (Figure 4) (Vanneste *et al.* 2013). Only 4% of *fhod-1(sb123)* arrested at this stage, indicating that *fhod-1(sb123)* can mediate elongation. As a more sensitive test, we examined genetic interactions with elongation pathway mutants. All *mel-11* embryos die at the restrictive temperature of 25° due to hypercontraction, but this can be suppressed by mutations such as *fhod-1(tm2363)* that lessen the contractile force (Vanneste *et al.* 2013). In particular, 53% of the embryos from *fhod-1(tm2363)*; *mel-11* hatched, with 13% surviving to L4/adult compared to 0% for *mel-11* controls (Figure 4, *P* < 0.0001). In contrast, *sb123* showed much weaker suppression of *mel-11*, with only 6% hatching and 3% growing to L4/adult. Nevertheless, this level of suppression is significant as hatched embryos represented 40/731 of the *fhod-1(sb123)*; *mel-11* progeny laid. In comparison, only 1/3026 of control *mel-11* embryos hatched (*P* < 0.0001). Together, the data indicate that *fhod-1(sb123)* retains substantial wild-type elongation function, implying that *fhod-1b(+)* primarily mediates elongation, with *fhod-1a(+)* playing a minor role.

Since total FHOD-1 would be decreased slightly in *fhod-1(sb123)* due to the loss of FHOD-1a, we asked if a simple 50% decrease in *fhod-1* function in heterozygotes could rescue *mel-11*. However, none of the embryos of *fhod-1(tm2363)/+*; *mel-11* hatched, indicating a lack of suppression (Figure 4, 1/564 hatched, *P* > 0.99).

As another assessment of *fhod-1a* function during elongation, we made double mutants with *fem-2*, which alone has impenetrant hypoelongation phenotypes. The null allele *fhod-1(tm2363)* strongly enhanced *fem-2* (Figure 4) (Vanneste *et al.* 2013), with only 19% growing to L4/adult compared to the values for the single mutants of 76% for *fhod-1* and 94% for *fem-2*. In contrast, 70% of *fhod-1(sb123)*; *fem-2* grew to L4/adult, again suggesting that *fhod-1(sb123)* retains substantial wild-type elongation function. Nevertheless, the genetic interactions of *fhod-1(sb123)* with *fem-2*, albeit much weaker than that for the null allele, was significant compared with the *fhod-1(sb123)* control (*P* < 0.0001). These data again indicate that the short isoform *fhod-1b* has the major role during elongation, with the long isoform *fhod-1a* playing an ancillary role.

**Figure 5:**
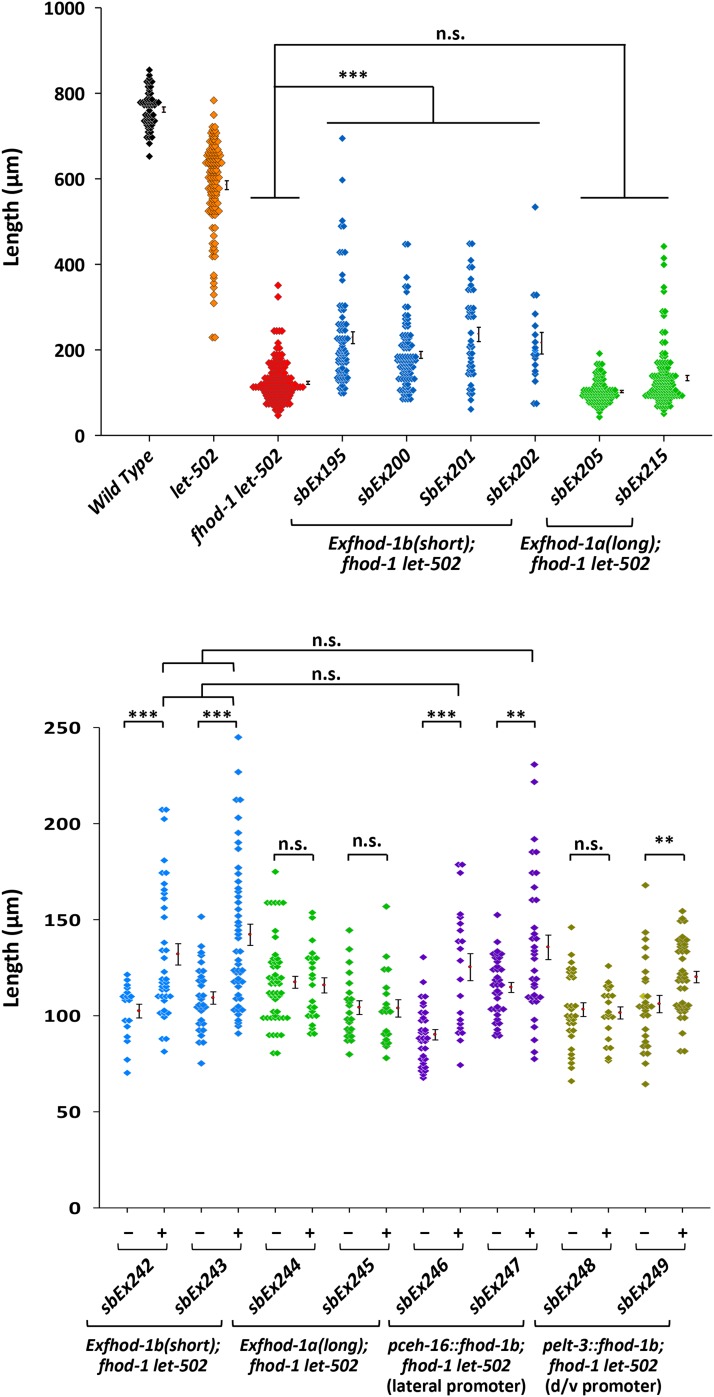
Transgenic rescue by *fhod-1* isoforms and tissue-specific rescue. Hermaphrodites were cultured at the semi-permissive temperature of 24° and progeny lengths of semi-synchronized broods were measured 2 days after egg laying (see MATERIALS and METHODS). The null *fhod-1(tm2363)* was sensitized with the *ts* allele *let-502(sb118)*. There was little overlap in lengths of *let-502* and *fhod-1 let-502* controls. (A) Independent extrachromosomal transgenes containing the native *fhod-1* promoter driving *fhod-1b* cDNA without exon 8 (*sbEx195*, *sbEx200*, *sbEx201* and *sbEx202*) resulted in far more animals overlapping control *let-502* lengths than did cDNAs encoding the longer isoform *fhod-1a* (*sbEx205*, *sbEx215*). (B) Transgenic lines marked with SUR-5::GFP enabled scoring of transgenic progeny in comparison to sibs who did not inherit the array. Rescue was seen among GFP+ progeny for the short (*sbEx242*, *sbEx243*) but not the long (*sbEx244*, *sbEx245*) *fhod-1* isoform under the control of the native *fhod-1* promoter. The short isoform was then driven using lateral-specific *pceh-16* or dorsal/ventral-specific *pelt-3* promoters. Rescue was seen for both lines using the lateral promoter (*sbEx247*, *sbEx248*) and was not significantly different from the control lines (*sbEx242*, *sbEx243*) that employed the native promoter. One dorsal/ventral line (*sbEx249*) did rescue, but none of the progeny fell outside the range of the control sibs. Unhatched embryos are ∼50 µm. Error bars = standard error of the mean. Because the lengths did not show normal distributions, the Kruskal-Wallis One Way Analysis of Variance on Ranks was used rather than ANOVA. *** = *P* < 0.001, ** = *P* < 0.01, n.s. = *P* > 0.05, not significant. Supplemental Figure 4 shows comparison of this method of assessing growth to scoring with the dissecting microscope used in Figures 2 and 4.

It is possible that the differing phenotypes for *fhod-1a* and *fhod-1b* stem not from the presence or absence of alternatively-spliced exon 8, but rather tissue-specific splicing to yield only *fhod-1b* in the epidermis. To test this, we created cDNA transgenics for the short and long form, driven by the endogenous *fhod-1* promoter. Because of the low penetrance phenotype of even the more severe *fhod-1(tm2363)* allele, we injected constructs into the sensitized *let-502(sb118) fhod-1(tm2363)* background. *let-502(sb118)* is a hypomorphic, *ts* mutation that strongly enhances the null *fhod-1(tm2363)* allele (Vanneste *et al.* 2013). Because transgenes usually do not show maternal expression (Kelly *et al.* 1997), we first confirmed that zygotic *fhod-1(+)* effectively rescued the strain (Figure S1B). As a sensitive, quantitative metric to assess rescue by the long and short *fhod-1* isoforms, gravid adults were brooded for 2-3 hr at the semi-permissive temperature of 24° and 1 or 2 days later body length was measured. We found that 2 days of growth amplified the differences between transgenic animals and the non-growing *fhod-1let-502* controls (Figure S3). Figure S4 shows that the more subjective scoring scheme using 24 hr cohorts, as presented in Figures 2 and 4, yielded comparable results to the of scoring 2-3 hr cohorts, as used in Figure 5. Slow growing/arrested larvae invariably had lumpy and dumpy body shapes characteristic of elongation mutants. Four independent *fhod-1b* transgenic lines showed substantially better growth than controls (*P* < 0.001), while two *fhod-1a* transgenic lines did not (Figure 5A). Thus, *fhod-1b*, but not *fhod-1a*, appears to be at least partially sufficient in the epidermis for elongation, consistent with the *fhod-1(sb123)* results.

Because of the mosaic nature of extrachromosomal transgenes, the incomplete rescue seen in Figure 5A could be due to some progeny of transgenic animals not inheriting the transgenes. To test this, we made transgenic lines in the *fhod-1let-502* background that included the ubiquitous marker *sur-5*::*gfp* (Gu *et al.* 1998) and compared GFP+ to GFP- progeny. Figure 5B again shows rescue by the short *fhod-1b* isoform but not by the long *fhod-1a*. For *fhod-1b*, the GFP+ progeny showed significantly more growth than their GFP- sibs (*P* < 0.001), but rescue was again incomplete. Unlike the rescued animals shown in Figure 5A, no GFP+ progeny in Figure 5B grew to >250 µm 2 days after laying, indicating that the *sur-5*::*gfp* marker may impede growth and/or transgene function.

We conclude from the loss-of-function experiments with the CRISPR generated *fhod-1(sb123)* mutant (Figure 4), and the transgenic rescue work (Figure 5A,B), that the short isoform *fhod-1b* is primarily responsible for elongation, with the long isoform *fhod-1a* playing a supplemental role.

### fhod-1 acts in lateral epidermal cells during elongation

We next asked where *fhod-1b* expression is sufficient to mediate elongation. We previously found *fhod-1* microfilaments defects only in lateral cells. Immunostaining with antibodies to FHOD-1 had highest expression in lateral cells, albeit expression was not detected until late in elongation, even though the impenetrant elongation arrest phenotype was visible by light microscopy during early elongation (Vanneste *et al.* 2013). To determine if *fhod-1* expression in lateral cells is indeed required for elongation, we drove the *fhod-1b* cDNA using drivers that are specifically active in lateral cells (*ceh-16*) or dorsal/ventral cells (*elt-3*). As shown in Figure 5B, lateral expression of *fhod-1b* partially rescued *fhod-1let-502* elongation (*P* < 0.001 for *sbEx246* and < 0.01 for *sbEx247*). Notably, the two rescuing lines were not significantly different from either of the two control *fhod-1b* lines driven by the native promoter (*P* > 0.1). One dorsal/ventral line (*sbEx248*) was not rescued while a second line (*sbEx249*) showed a slight increase in mean body length (*P* < 0.01). However, unlike other rescued lines in Figure 5, there were no animals above the control range. We also created one transgenic line that did not contain *sur-5*::*gfp*, and this did not show rescue (Figure S4). Thus, lateral expression appears to be more important for elongation, although dorsal/ventral *fhod-1* may play a secondary role.

### fhod-1(sb123) indicates that the long isoform functions in muscle

**Figure 6:**
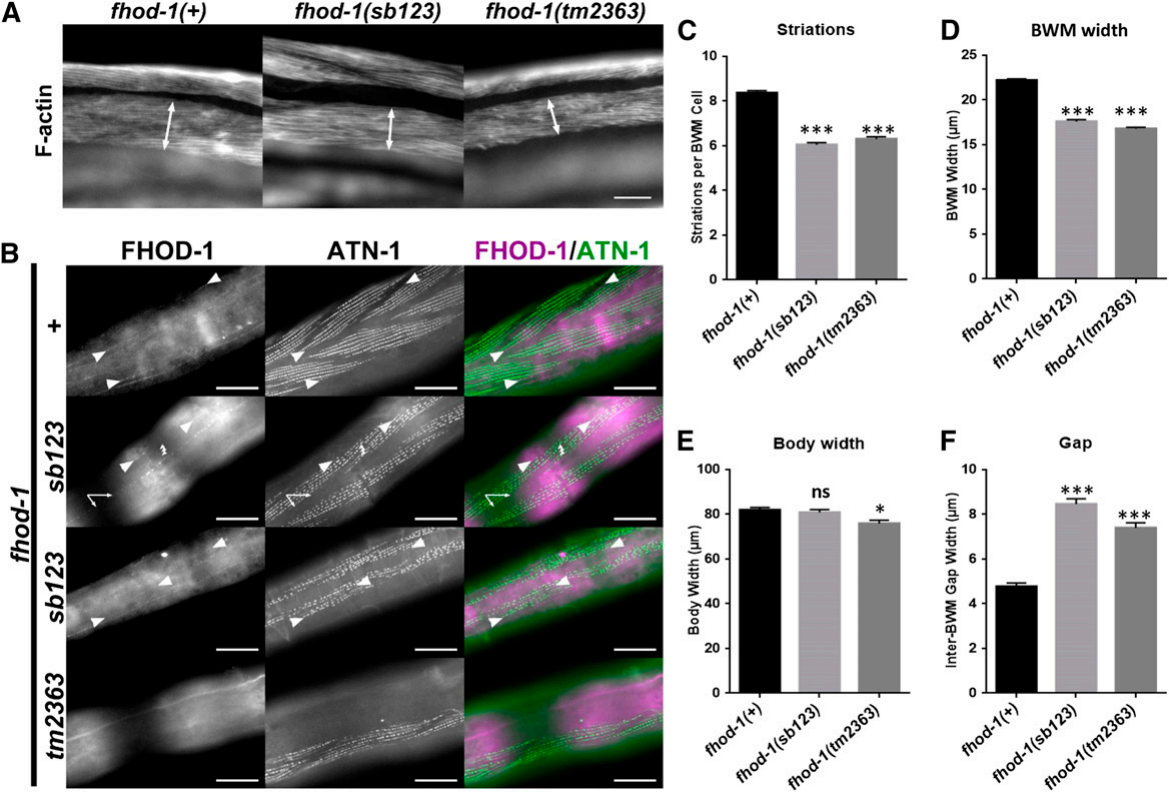
Muscle defects in *fhod-1(sb123)*, which encodes only the short isoform, are as severe as the null allele *fhod-1(tm2363)*. (A) Fluorescent phalloidin-stained F-actin in young adult stage animals. The arrow indicates the width of body wall muscles (BWM), which have fewer (C) and narrower (D) striations in both *sb123* and *tm2363*. (B) L4 larva stage animals double-stained for FHOD-1 and Z-line marker ATN-1, showing that FHOD-1 is expressed in puncta along the edges of F-actin–rich body wall muscles cell contractile lattices (large arrowheads) and in faint striations across the lattices (small arrowheads). FHOD-1 levels are reduced in *fhod-1(sb123)* and absent in *fhod-1(tm2363)*. The small arrows indicate non-specific stain by anti-FHOD-1 noted previously (Mi-Mi *et al.* 2012). Body width (E) was similar to wild type in both mutants while the gap between muscles (F) was similarly increased in both alleles. *t*-tests of mutants relative to wild type, * *P* ≤ 0.05, *** *P* ≤ 0.001, ns = *P* > 0.05, not significant. Scale bar, 100 µm.

We asked if the two *fhod-1* isoforms differ in their muscle function. Wild-type body-wall muscle (BWM) cells assemble 8-10 striations from embryogenesis through young adulthood. In null *fhod-1(tm2363)*, addition of BWM striations through larval development is slower than wild type, resulting in only 6-7 striations per cell, and narrow BWM (Mi-Mi *et al.* 2012). Staining of F-actin in *fhod-1(sb123)* animals with fluorescent phalloidin showed that these mutants are nearly indistinguishable from *fhod-1(tm2363)* animals in terms of having fewer striations and narrower BWM than wild type (*P* < 0.001, Figure 6A,C,D). This was not a reflection of overall reduction in body size, as there were only modest differences in body width between wild-type and *fhod-1(sb123)* worms, although *fhod-1(tm2363)* animals were slightly narrower (Figure 6E).

Thin filaments in BWM cells are anchored primarily to dense bodies that are rich in α-actinin/ATN-1 (Moulder *et al.* 2010). In wild-type muscle, dense bodies are evenly distributed along striations, while in *fhod-1(tm2363)* muscle, dense body spacing is irregular (Mi-Mi *et al.* 2012). In *fhod-1(sb123)* muscle, dense bodies are also irregularly spaced, similar to *fhod-1(tm2363)* (Figure 6B). Thus, in terms of BWM cell morphology, the effects of *fhod-1(sb123)* closely resemble those of FH2-disrupting *fhod-1(tm2363)*. This implies that the *fhod-1a* long isoform, which is deficient in *fhod-1(sb123)*, is required in muscle. In wild type, FHOD-1 localizes in BWM from mid-larval development through young adulthood, concentrating along the lateral edges of muscle cells (Figure 6B, large arrowheads), with a much fainter, diffuse presence along striations (small arrowheads). FHOD-1 was also visible in these locations in *fhod-1(sb123)* BWM cells, but much more faintly, and less consistently. Thus, endogenous FHOD-1B has a limited capacity to assemble but apparently is not sufficient for normal morphology.

FHOD-1 is also required for the proper positioning of BWM. During embryogenesis, BWM cells migrate from lateral positions to the dorsal or ventral surfaces, where they form dorsal or ventral pairs of muscle. The gap between muscles in each pair are wider in *fhod-1(tm2363)* worms than in wild-type animals, suggesting mutant BWM cells do not migrate the full distance from their lateral positions during embryogenesis (Mi-Mi *et al.* 2012). In *fhod-1(sb123)* mutants, the gap not only is wider than wild-type, but surprisingly, is consistently wider than in *fhod-1(tm2363)* mutants (Figure 6B, F).

To test whether the defective BWM phenotype of *fhod-1(sb123)* worms reflects just a partial reduction in FHOD-1 expression levels, we compared BWM of wild-type animals to those heterozygous for *fhod-1(tm2363)*. Among three replicated experiments, there were no consistent differences in BWM width, number of striations in individual BWM cells, or in width of the gaps between BWMs (data not shown).

### The short isoform can function in muscle when expressed as a transgene

**Figure 7:**
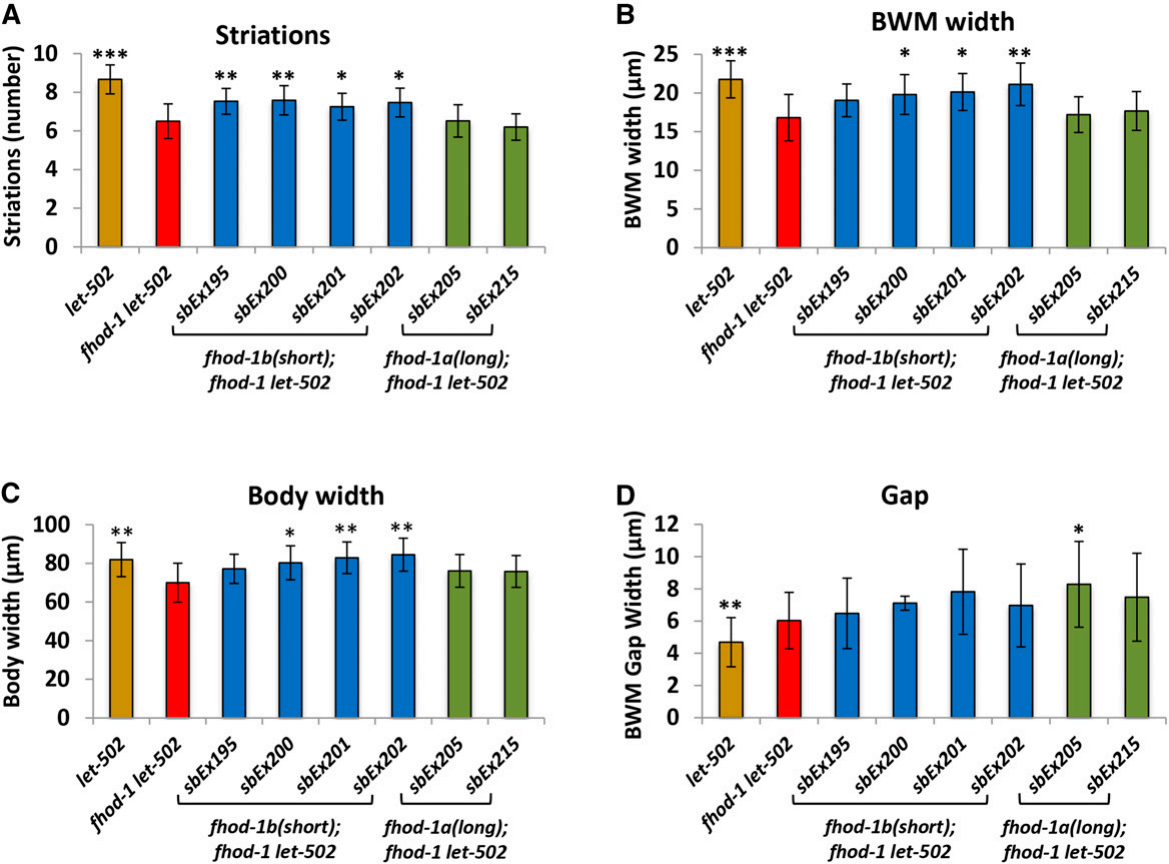
Transgenic rescue of the muscle defects by *fhod-1a* and *fhod-1b*. The same transgenic lines used in Figure 5A were scored for muscle phenotypes and are color coded similarly in the two figures. Extrachromosomal transgenes containing the native *fhod-1* promoter driving *fhod-1* cDNA without exon 8 (*sbEx195*, *sbEx200*, *sbEx201* and *sbEx202*) rescued most *fhod-1 let-502* muscle defects. cDNAs encoding the longer isoform *fhod-1a* (*sbEx205*, *sbEx215*) for the most part did not rescue. (A) striations, (B) body wall muscle width, (C) body width, (D) gap between muscle cells.

While the results from the *fhod-1(sb123)* allele showed that the long isoform is necessary for muscle function, transgenic expression of only the short *fhod-1b* isoform was sufficient to at least partially rescue some muscle phenotypes. Using the same transgenic strains used in Figure 5 to drive the long and short isoforms by the native *fhod-1* promoter, muscle striations, BWM width and body width were often rescued by the short isoform *fhod-1b* (Figure 7A-C). Surprisingly, the long isoform was not sufficient to rescue these phenotypes. Additionally, transgenics for neither isoform were not able to rescue the muscle gap phenotype (Figure 7D). These data may indicate that expression levels in muscle, in addition to (or rather than) the coiled-coil region present in *fhod-1a*, are responsible for the *fhod-1(sb123)* muscle phenotypes (see Discussion).

### Redundancy of fhod-1 with other formins or actin nucleators

The incomplete penetrance of *fhod-1* single mutant phenotypes suggests redundancy with other genes that regulate actin nucleation for elongation. Thus, we examined the other *C. elegans* formins as well as genes from the Arp2/3 actin nucleation pathway for elongation defects and genetic interaction with *fhod-1*. All analyses were carried out at 25° and putative formin strong to null alleles were used (Mi-Mi *et al.* 2012). Double mutants of *fhod-1* and a gene acting in parallel would show synergistic genetic interactions, but as described below, we did not detect any.

*C. elegans* has six other formin family genes (see Introduction). *exc-6* was the best candidate for acting in parallel with *fhod-1* as *exc-6* is the only formin mutant that exhibited an early elongation arrest similar to that of *fhod-1*, as shown by microscopic analysis of the 20% of embryos that did not hatch. Additionally, *fhod-1*; *exc-6* adults often show a rolling phenotype (Mi-Mi *et al.* 2012), which is characteristic of weak *let-502* alleles (Piekny *et al.* 2000). However, frequencies of unhatched embryos and elongation arrested larvae of *fhod-1*; *exc-6* appeared additive rather than synergistic (Figure S6). Because *cyk-1* acts in parallel to *fhod-1* in muscle (Mi-Mi *et al.* 2012; Mi-Mi and Pruyne 2015), we examined the double mutant for elongation defects, but again the interactions appeared additive rather than synergistic (*cyk-1* is homozygous maternal-effect lethal and so *cyk-1/+*; *fhod-1* was examined). RNAi knockdown of *let-502*, which enhances *fhod-1* (Vanneste *et al.* 2013), was used to further sensitize *fhod-1*; *exc-6* and *fhod-1*; *cyk-1/+* but showed only additive enhancement (Figure S6, combinations of *let-502(RNAi)* and *cyk-1/+* did result in high percentages of unhatched embryos, but these may stem from the requirement of both genes for cytokinesis in the early embryo (Swan *et al.* 1998; Piekny and Mains 2002)). We extended the analysis to triple formin knockdowns. Treatment of the *fhod-1*; *exc-6* with RNAi to *fozi-1*, *daam-1* or *cyk-1* had no synergistic effects (Figure S7). Similarly, *let-502(RNAi)* feeding of **inft-2(ok1296)**, *frl-1(ok460)*, *fozi-1(ok1182)* and *exc-6(gk386)* mutants had additive rather than synergistic interactions, suggesting that these formins do not function in the *let-502* elongation pathway.

The ARP2/3 complex may potentially act in parallel to formins to nucleate microfilaments during elongation. We examined the elongation phenotype of the two Arp2/3 complex activators WVE-1/WAVE and WSP-1/WASP (Sawa *et al.* 2003). *let-502(RNAi)* had little effect on those two strains (Figure S7).

### The role of muscle cells in early elongation

**Figure 8:**
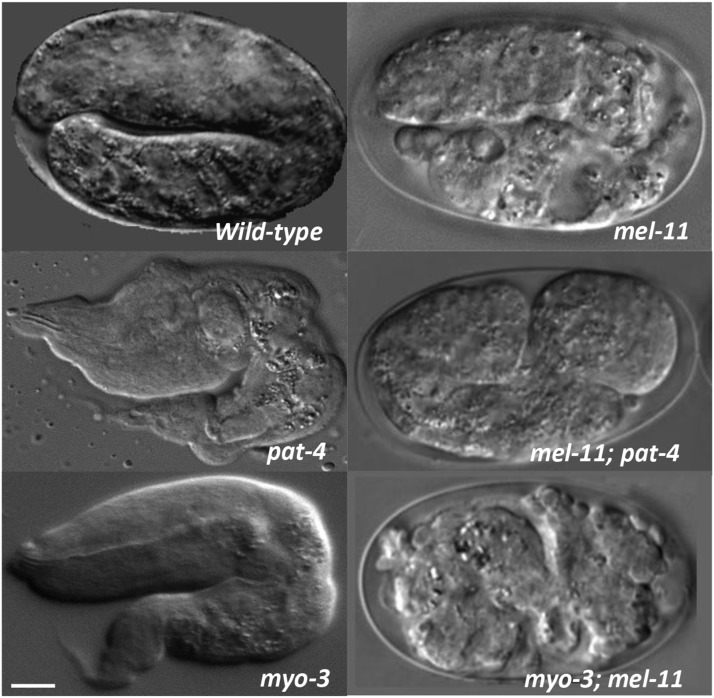
Muscle mutants do not suppress the *mel-11* hypercontraction defect. Top row, wild type is comparted to *mel-11*, which shows a disorganized embryo, with posterior cells not encased in the epidermis in this particular embryo. Middle row, *pat-4* arrests at 2-fold while *pat-4*; *mel-11* shows the *mel-11* phenotype. Bottom row, *myo-3* arrests at 2-fold while *myo-3*; *mel-11* shows the *mel-11* phenotype. Experiments were done at 25°C and anterior is to the left and dorsal to the top in all images. Scale bar = 10 µm.

Early elongation is redundantly controlled by the *let-502/mel-11* and *fem-2/pak-1* pathways, while late elongation (after the 2-fold stage) depends on muscle contraction. Since *pak-1* and its activator *pix-1* function in muscle/epidermal attachments during late elongation and *fhod-1* functions in muscle formation (Zhang *et al.* 2011; Mi-Mi *et al.* 2012), it is formally possible that muscle function also has a role in early elongation (*i.e.*, in parallel to let-502/mel-11). To test this possibility, we took advantage of the fact that mutations in genes acting during early elongation (*e.g.*, *let-502, fem-2, fhod-1*) suppress the hypercontraction phenotype of *mel-11*. We asked if compromising muscle function will act similarly to rescue *mel-11*. Mutations in muscle genes encoding two biochemically different products were tested for *mel-11* suppression. *pat-4*/integrin-linked kinase (Mackinnon *et al.* 2002) encodes a focal adhesion protein necessary for muscle development and *myo-3* encodes myosin heavy chain A (Miller *et al.* 1986). Despite their relatively late phenotypes (Pat, paralyzed arrest at two-fold stage), transcripts of both genes are detected earlier (Hashimshony *et al.* 2015). If mutations of either gene suppress *mel-11*, progeny of the double mutants should exhibit the Pat phenotype rather than arresting due to the hypercontraction of *mel-11*. *mel-11*; *pat-4/+* embryos all exhibited the *mel-11* hypercontraction phenotype (n = 63), rather than the expected 25% Pat phenotype (Figure 8). Similarly, the hypercontraction phenotype was epistatic to Pat among the progeny of *mel-11(it26);* *myo-3/+* (n = 100). Additionally, *myo-3* did not enhance early elongation defects of *let-502*(RNAi) (data not shown), again indicating that *myo-3* does not mediate early elongation. As shown earlier, driving *fem-2* cDNA in BWM cells did not rescue the *fem-2* elongation phenotype (Figure 2). In conclusion, we found no evidence for a role of muscle cells during the early phase of elongation.

## Discussion

Morphogenesis within a population of cells is carried out with exquisite precision, with individual cells often implementing different subprograms. For example, some cells may remain rigid, while adjacent cells change their forms, or cells may assume complementary shapes. The actomyosin mediated elongation of the *C. elegans* embryo from a spheroid into a tube is a simple morphogenetic model that displays these properties. The *C. elegans* epidermis has two general cell types – lateral cells provide the primary contractile force while dorsal and ventral cells play a more passive role (Priess and Hirsh 1986; Diogon *et al.* 2007; Gally *et al.* 2009; Vuong-Brender *et al.* 2017). These differences may be in part mediated by components of the two parallel elongation pathways acting primarily in either lateral or dorsal/ventral cells. For example, previous transgenic experiments showed that RHGF-2/Rho GEF can rescue mutants when expressed laterally while RGA-2/Rho GAP acts dorsal/ventrally (Diogon *et al.* 2007; Chan *et al.* 2015). Based on phenotypic analysis, PIX-1/CDC42-RAC GEF and PAK-1/ p21 activated kinase have dorsal/ventral roles (Martin *et al.* 2014; Martin *et al.* 2016). It should be kept in mind that genes may not be entirely on or off in different cells types, but rather differ by high or low levels of activity, which could refine morphogenic changes. Here we have described additional genes with primarily lateral or dorsal/ventral functions: FEM-2/PP2c phosphatase can act dorsal/ventrally and the FHOD-1/Formin functions in the lateral cells.

### FEM-2 primarily acts in the dorsal/ventral epidermis while FHOD-1 acts laterally

Based on transgenic rescue experiments, FEM-2 expression in the dorsal/ventral epidermal cells is at least partially sufficient to provide elongation function (Figure 2). Rescue by dorsal/ventral expression in the sensitized *fem-2(-)*; *fhod-1(-)* background was not to the levels seen in *fem-2(+)*; *fhod-1(-)*, likely due to inefficient and/or mosaic transgene expression. However, rescue was comparable to the *fem-2* transgene expressed by its native promotor. In dorsal/ventral epidermal cells, *fem-2* likely interacts with *pak-1* and *pix-1* (Martin *et al.* 2014; Martin *et al.* 2016), which would be consistent with genetic interactions (Vanneste *et al.* 2013). In mammals, biochemical assays show that PP2C phosphatase, the FEM-2 homolog, is a regulator of PAK1 (Chan *et al.* 2008).

*fhod-1* appears to act laterally, partially rescuing the sensitized *fhod-1let-502* background (Figure 5). This is consistent with *fhod-1* genetic interactions with the laterally active *let-502* (Vanneste *et al.* 2013). Similar to what we observed with *fem-2*, *fhod-1* rescue was incomplete, although lateral-only rescue was not statistically different from that of the native promoter. Incomplete rescue may reflect inherent limitations of transgenic gene expression. The *ceh-16* and *elt-3* promoters used here, as well in other studies of elongation gene function, typically result in incomplete rescue (Diogon *et al.* 2007; Gally *et al.* 2009; Lin *et al.* 2012; Chan *et al.* 2015), possibly because the expression levels and/or timing vary from that of the endogenous promoters. Although lateral *fhod-1* expression is clearly most important during elongation, weak rescue was seen with one of three dorsal/ventral lines (*sbEx249*, Figure 5, Figure S5). Average length increased without increasing the length of any animals beyond the control range. This may indicate that while lateral *fhod-1* function is most important, dorsal/ventral activity could provide supplemental function. Similarly, although expression of *fem-2* in lateral epidermal cells or body wall muscle cells failed to rescue *fem-2*; *fhod-1* (Figure 2), these tissues might supplement dorsal/ventral *fem-2* mediated elongation to refine the process.

### FHOD-1 isoforms may have specialized functions

FHOD-1 has two isoforms that may be partly specialized for epidermal or muscle function. Formin family proteins act by nucleation and bundling of linear actin filaments (Breitsprecher and Goode 2013; Bechtold *et al.* 2014; Kuhn and Geyer 2014; Pruyne 2016) and different functional FHOD isoforms (in different parts of the protein than we report here) are known in other species (Kan-O *et al.* 2012b; Iskratsch *et al.* 2013). Previously we showed that *fhod-1* acts in the *let-502* branch of the elongation pathway (Figure 1) as well as in striated muscle (Mi-Mi *et al.* 2012; Vanneste *et al.* 2013; Mi-Mi and Pruyne 2015). Here we found that *fhod-1* encodes two isoforms that differ in the inclusion of the 474 bp exon 8, which includes a predicted coiled-coil in the longer *fhod-1a* allele. To explore functional differences between the predicted proteins, we created a CRISPR allele (*sb123*) that eliminates only the longer isoform. The results from *fhod-1(sb123)* showed that *fhod-1b* expression is primarily responsible for elongation: while the likely null allele *fhod-1(tm2363)* shows strong genetic interactions with *mel-11* and *fem-2* (Vanneste *et al.* 2013), these interactions were substantially weaker for *sb123* (Figure 4). However, while weak, the genetic interactions were statistically significant, indicating the long isoform has a supplementary role during elongation. In muscle, the opposite pattern was seen, with *fhod-1(sb123)* and *fhod-1(tm2363)* showing similar phenotypes (Figure 6). Based on the mutant alleles, the short form is more important for elongation, while the long form is primarily required in muscle. Transgenic rescue, which demonstrates sufficiency rather than necessity, showed more complicated results. As expected from the *fhod-1(sb123)* data, transgenic expression of the short, but not the long form, partially rescued elongation (Figure 5). Unexpectedly, the short form at least partially rescued muscle phenotypes (Figure 7). While low levels of FHOD-1b were able to assemble into muscle in *fhod-1(sb123)* (Figure 6B), this was not sufficient to rescue. Perhaps overexpression, which commonly occurs for multicopy transgenes, leads to FHOD-1b muscle rescue. Why potential overexpression of FHOD-1a long form is not sufficient during either elongation or muscle formation is not clear. Because of the mosaic expression of transgenes, the difficulty in synchronizing large numbers of embryos, and the inability to distinguish between expression in different tissues, we did not measure protein levels by Western blots in transgenic strains. It is possible that the *fhod-1(sb123)* results from differences in isoform expression levels of the endogenous gene rather than differences in protein isoforms as *fhod-1b* is expressed at higher levels than *fhod-1a*, (Figure 3C). Arguing against this was the lack of elongation or muscle phenotypes when *fhod-1* levels were decreased by 50% in heterozygotes (Figure 4 and data not shown).

*fhod-1* elongation defects are incompletely penetrant ((Vanneste *et al.* 2013), Figure 4), suggesting redundancy with other actin nucleators. Testing mutants of the other six formin genes (*daam-1*, *exc-6*, *inft-2*, *frl-1*, *fozi-1*, and *cyk-1*) encoded by the *C. elegans* genome showed no dramatic genetic interactions with *fhod-1* or *let-502* (Supplemental Figures 6 and 7). Only two of these genes, *exc-6* and *cyk-1*, had, at best, weak additive effects on *fhod-1* elongation arrest rather than synergistic interactions predicted for genes acting in parallel. Sensitizing the background further with *let-502* or testing triple formin mutants failed to detect strong interactions. These data indicate that *fhod-1* may be involved in “fine tuning” rather than mediating an essential process, in concert with other formins. Alternatively, the FHOD family actin bundling activity (Schonichen *et al.* 2013; Bechtold *et al.* 2014; Schulze *et al.* 2014) may act in conjunction with unknown proteins. While our data found no evidence for redundancy, a caveat of this work is that RNAi and use of heterozygous strains for lethal mutations could have resulted in insufficient knockdown to detect genetic interactions.

### Muscle genes are not involved in early elongation

*pak-1* (Gally *et al.* 2009; Vanneste *et al.* 2013) and *pix-1* mediate early elongation in parallel to *let-502*. Since *pak-1* and *pix-1* have roles in the attachments of muscle to the epidermis that are required during late elongation (Zhang *et al.* 2011), we asked if muscle has a previously unknown role during early elongation in addition to its later function after the 2-fold stage. However, epistasis experiments (Figure 8) indicated that muscle is likely not involved in early elongation, since compromising muscle does not block the early *mel-11* hypercontraction phenotype.

### Conclusion

Lateral and dorsal/ventral cells have different roles during elongation, and this is reflected by different genetic requirements. However, while wild-type expression in one cell type might not be strictly necessary for successful elongation, that expression may fine tune the final form or lend robustness to the process. It is likely that many levels of redundancy are involved in elongation.
